# Supplementary material for: p97/VCP is required for piecemeal autophagy of aggresomes
Source: Nat Commun. 2025 May 7;16:4243. doi: 10.1038/s41467-025-59556-x (PMC12059050; doi:10.1038/s41467-025-59556-x)
Supplement: Supplementary file 1 — Supplementary Information [file 41467_2025_59556_MOESM1_ESM.pdf]

Supplementary Information

p97/VCP is required for piecemeal autophagy of aggresomes

Maria Körner[1,5], Paul Müller[1,5], Hirak Das[2], Felix Kraus[3], Timo Pfeuffer[1], Sven Spielhaupter[1], Silke Oeljeklaus[2], Christina Schülein-Völk[4], J. Wade Harper[3], Bettina Warscheid[2] and Alexander Buchberger[1,*]

[1] University of Würzburg, Biocenter, Chair of Biochemistry I, Am Hubland, 97074 Würzburg, Germany

[2] University of Würzburg, Biocenter, Chair of Biochemistry II, Am Hubland, 97074 Würzburg, Germany

[3] Department of Cell Biology, Harvard Medical School, Boston, Massachusetts 02115, USA

[4] University of Würzburg, Biocenter, Core Unit High-Content Microscopy, Am Hubland, 97074 Würzburg, Germany

[5] equal contribution

* Correspondence: alexander.buchberger@uni-wuerzburg.de

Contents:

Supplementary Figures 1–8

Supplementary Table 1

Supplementary References

Supplementary Figures

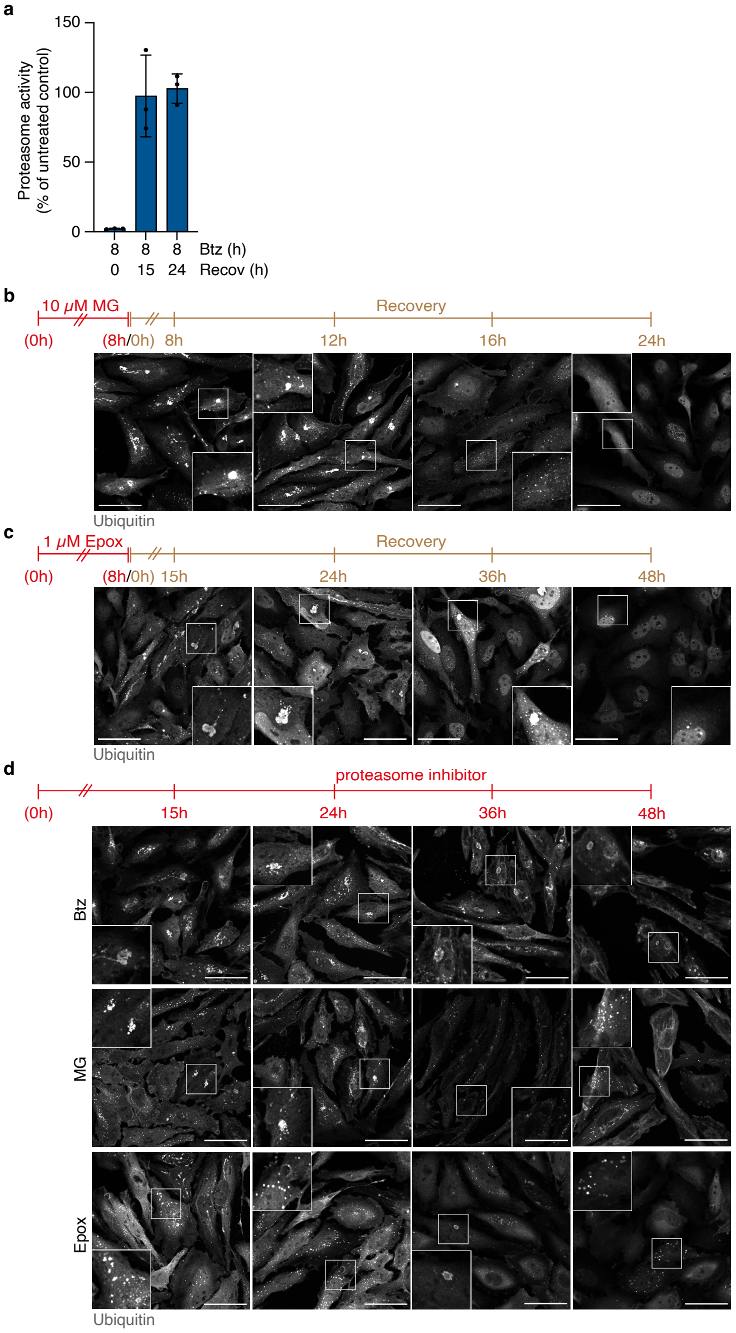

Supplementary Fig. 1 | Proteasome activity after Btz treatment and comparison of proteasome inhibitors.
a Proteasome activity was determined using an AMC-tagged peptide substrate in HeLa cells recovering for 0, 15 or 24h from Btz treatment and normalized to untreated control cells. Shown is the mean ± SD from n = 3 biological replicates with ≥ 8000 cells quantified per replicate and time point. **b, c** HeLa cells were treated for 8h with 10 µM MG-132 (**b**) or 1 μM Epoxomicin (**c**), recovered for 8 to 48h and analyzed by confocal immunofluorescence microscopy using antibodies against ubiquitin. Scale bars, 50 µm. Representative results from two independent experiments. **d** HeLa cells were treated constantly with 0.5 μM Btz, 5 µM MG-132 or 0.5 μM Epoxomicin and analyzed as in **b**, **c**. Scale bars, 50 µm. Representative result from two independent experiments.

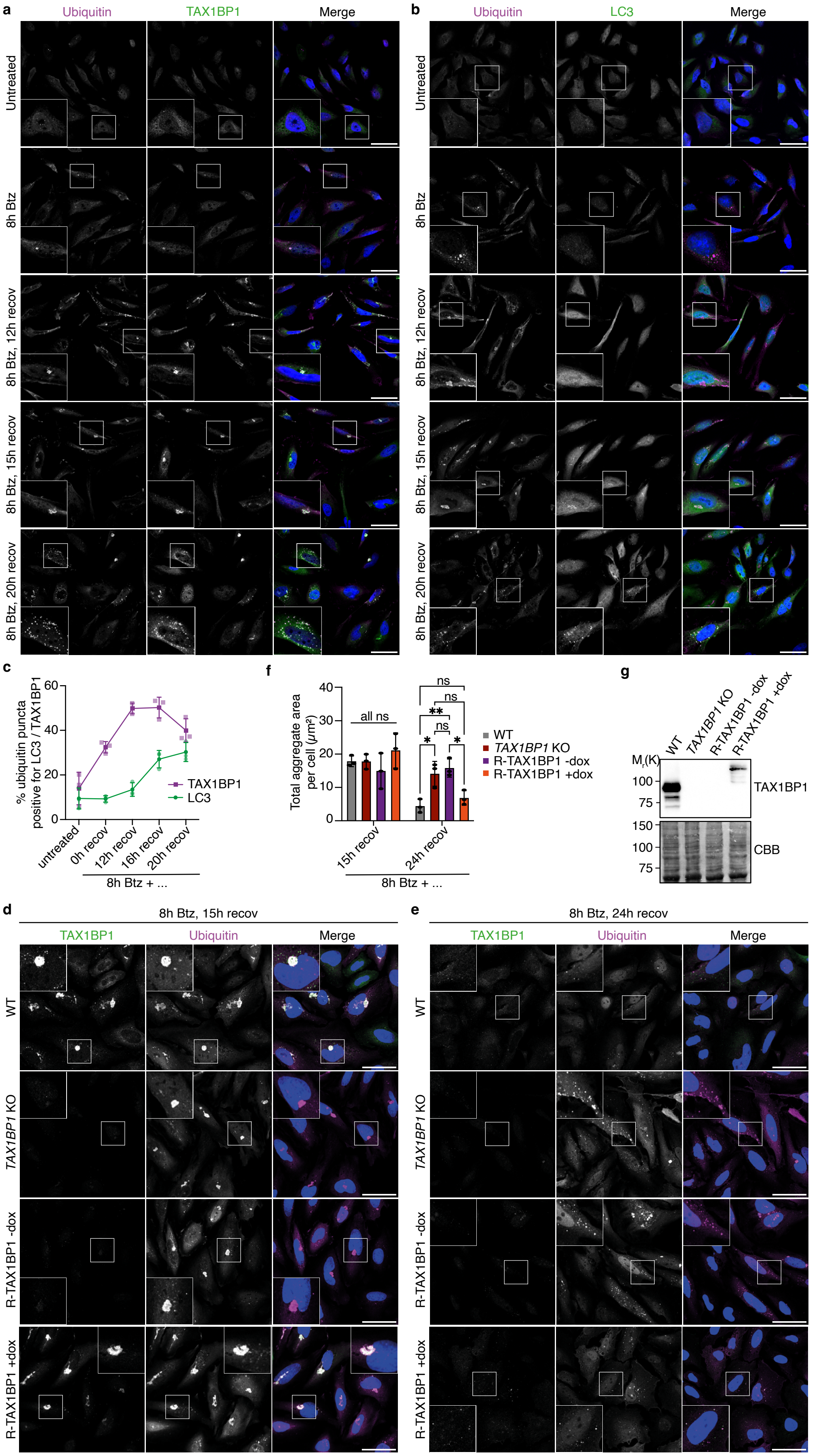

Supplementary Fig. 2 | Kinetics of TAX1BP1 and LC3 recruitment to aggregates/aggresomes and rescue of TAX1BP1 knock-out cells. **a, b** Recruitment of TAX1BP1 (**a**) and LC3 (**b**) to ubiquitylated aggregates in HeLa cells recovering for 0 to 24h from Btz treatment was analyzed by confocal immunofluorescence microscopy. Scale bars, 50 µm. **c** Quantification of the percentage of ubiquitin puncta positive for TAX1BP1 or LC3 in **a** and **b**, respectively. Shown is the mean ± SD from n = 3 biological replicates with ≥ 50 cells per time point and replicate. Individual data points are indicated by lighter colors. **d, e** HeLa wildtype (WT), *TAX1BP1* KO and *TAX1BP1* KO cells ectopically expressing TAX1BP1-mScarlet under the control of a doxycycline (dox)-inducible promoter (R-TAX1BP1) were treated with Btz (1 µM, 8h), followed by 15h (**d**) or 24h (**e**) recovery and immunostaining against TAX1BP1 and ubiquitin. Scale bars, 50 *µ*m. **f** Quantification of the total aggregate area per cell in **d** and **e**. Shown is the mean ± SD from n = 3 biological replicates with ≥ 37 cells per condition and replicate. Two-way ANOVA. **g** Lysates of cells described in **d, e** were analyzed by immunoblotting against TAX1BP1; the Coomassie stained membrane served as loading control (CBB). Representative result from two independent experiments.

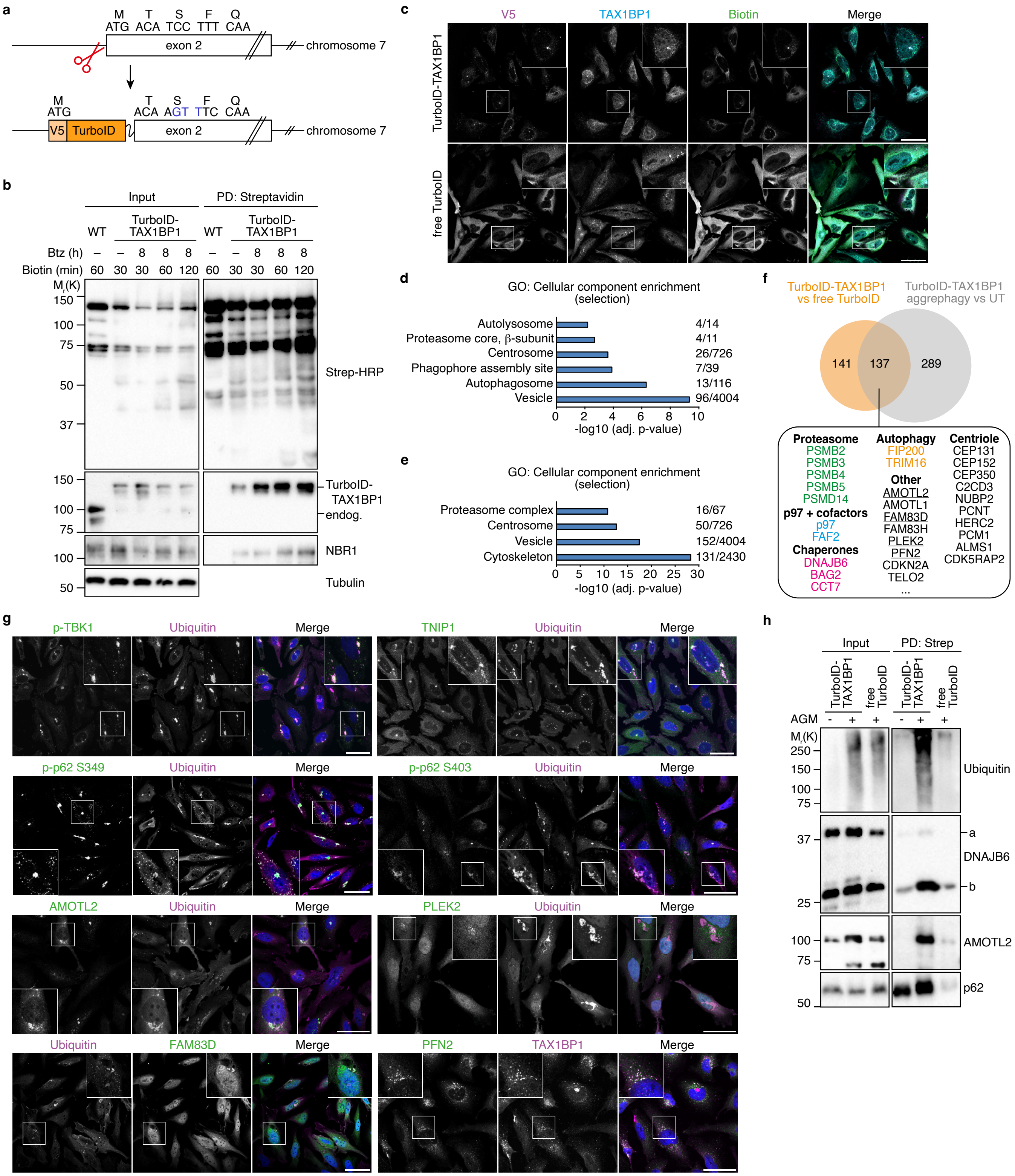

Supplementary Fig. 3 | Validation of the HeLa TurboID-TAX1BP1 knock-in cell line and the TAX1BP1 proximitome.
a Scheme illustrating the generation of a TurboID-TAX1BP1 expressing HeLa knock-in cell line *via* CRISPR-Cas9 based genome editing. **b** HeLa control (WT) or TurboID-TAX1BP1 knock-in cells were treated with Btz (1 µM, 8h) or left untreated, and 50 µM biotin was added in the last 30 to 120 min. After lysis, biotinylated proteins were enriched using streptavidin magnetic beads, and the input and pulldown (PD) fraction was immunoblotted using the indicated antibodies. Representative result from three independent experiments. **c** Confocal immunofluorescence microscopy of HeLa cells expressing TurboID-TAX1BP1 or free TurboID, treated with Btz (1 µM, 8h) in the presence of 100 nM BafA1 for the last 3h and 50 µM biotin for the last 1h, using the indicated antibodies. Scale bars, 50 µm. Representative result from four independent experiments. **d** Gene Ontology (GO) term enrichment of cellular components (selection) of TurboID-TAX1BP1 proximity partners significantly enriched over free TurboID proximity partners using g:Profiler. Ratios indicate the number of enriched proteins relative to the total number of proteins belonging to the respective component. **e** As in **d**, but for TurboID-TAX1BP1 proximity partners enriched under aggrephagy versus control conditions. **f** Venn diagram highlighting the overlap between significantly enriched proximity partners (log_2 FC > 1, adj. p-value < 0.05) in Fig. 2cd. Selected proteins are listed and colored according to the color code used in Fig. 2cd. Candidate cargo proteins characterized in this study are underlined. **g** Aggresome/aggrephagosome localization was validated as in Fig. 2e for additional TAX1BP1 proximity partners. Scale bars, 50 µm. Representative result from two independent experiments. **h** HeLa cells expressing TurboID-TAX1BP1 or free TurboID were treated as depicted in Fig. 2b. After lysis, biotinylated proteins were enriched using streptavidin magnetic beads, and the input and pulldown (PD) samples were immunoblotted against ubiquitin, DNAJB6 (isoforms a and b), AMOTL2 and p62; AGM, aggrephagy condition. Representative result from four independent experiments.

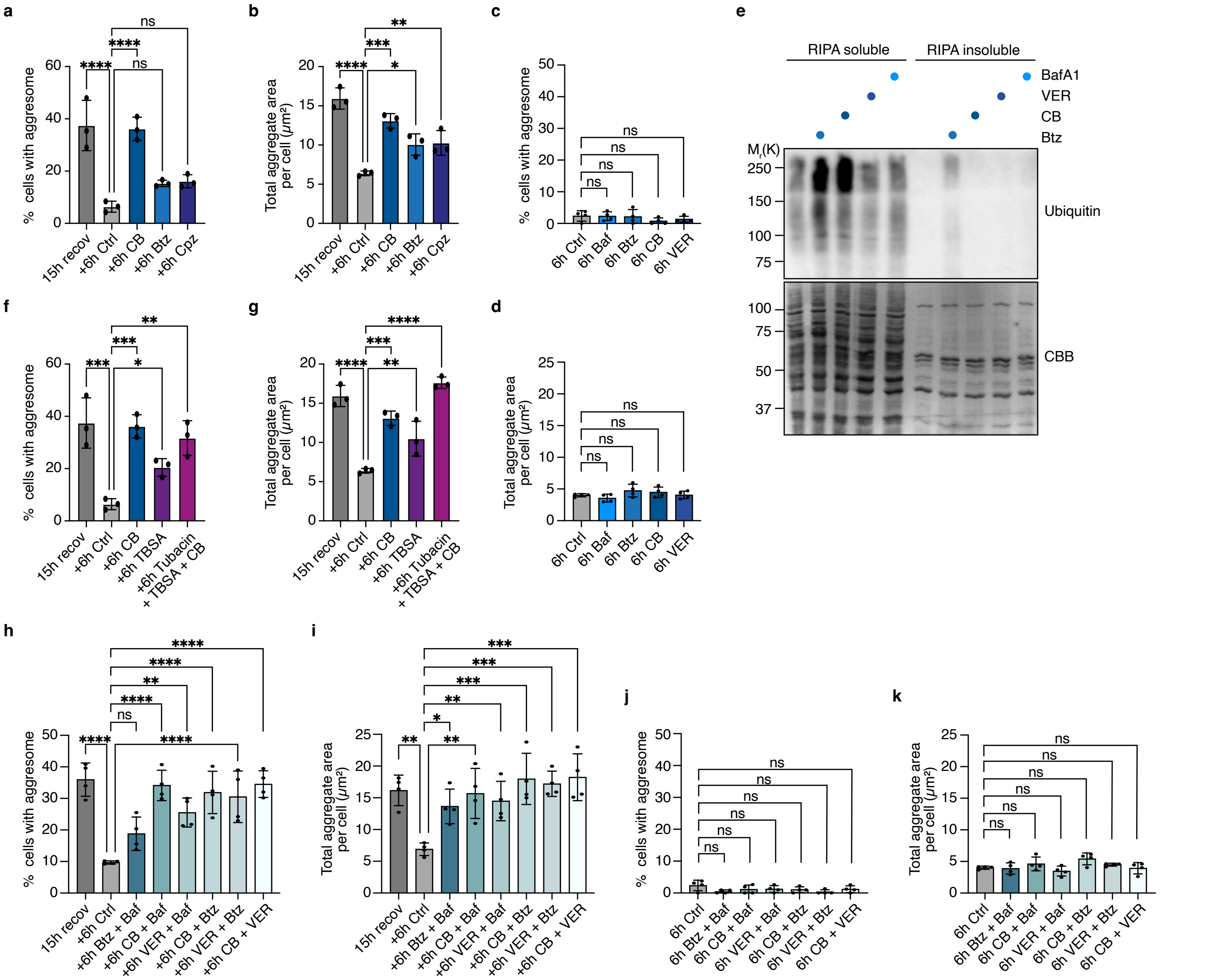

Supplementary Fig. 4 | Aggresome clearance upon combined inhibitor treatments.

a, b HeLa cells treated with Btz (1 µM, 8h) were fixed after 15h recovery or recovered for six additional hours in the absence (Ctrl) or presence of 1 μM CB-5083, 1 μM Btz or 10 μM Capzimin (Cpz), immunostained against ubiquitin and LC3 and analyzed by confocal microscopy. Quantification of the percentage of cells with an aggresome (**a**) and of total aggregate area per cell (**b**). Shown is the mean ± SD from n = 3 biological replicates with ≥ 60 cells per condition. One-way ANOVA. **c, d** HeLa cells were treated for 6h with 100 nM BafA1, 1 µM Btz, 1 µ M CB-5083 or 10 µM VER prior to fixation, immunostained against ubiquitin and LC3, and analyzed by confocal microscopy. Quantification of the percentage of cells with an aggresome (**c**) and of total aggregate area per cell (**d**). Shown is the mean ± SD from n = 3 biological replicates with ≥ 73 cells per condition. **e** HeLa cells treated as in **c** were lysed in RIPA buffer. The RIPA soluble and insoluble fractions were immunoblotted for ubiquitin, and Coomassie staining of the membrane served as loading control (CBB). Representative result from four independent experiments. **f, g** HeLa cells treated with Btz (1 µM, 8h) were fixed after 15h recovery or recovered for six additional hours in the absence (Ctrl) or presence of 1 μM CB-5083, 10 μM Tubastatin A (TBSA) or 10 μM Tubacin, prior to fixation, immunostaining against ubiquitin and LC3 and analysis by confocal microscopy. Quantification of the percentage of cells with an aggresome (**f**) and of total aggregate area per cell (**g**). Shown is the mean ± SD from n = 3 biological replicates with ≥ 54 cells per condition. One-way ANOVA. **h, i** HeLa cells treated with Btz (1 µM, 8h) were fixed after 15h recovery or recovered for six additional hours in the absence or presence of the indicated inhibitor combinations, followed by immunostaining against ubiquitin and LC3 and analysis by confocal microscopy. Quantification of the percentage of cells with an aggresome (**h**) and of total aggregate area per cell (**i**). Shown is the mean ± SD from n = 4 biological replicates with ≥ 74 cells per condition. One-way ANOVA. **j, k** HeLa cells treated with the indicated inhibitor combinations for 6h without prior Btz treatment were immunostained for ubiquitin and LC3. Quantification of the percentage of cells with an aggresome (**k**) and of total aggregate area per cell (**j**). Shown is the mean ± SD from n = 4 biological replicates with ≥ 73 cells per condition.

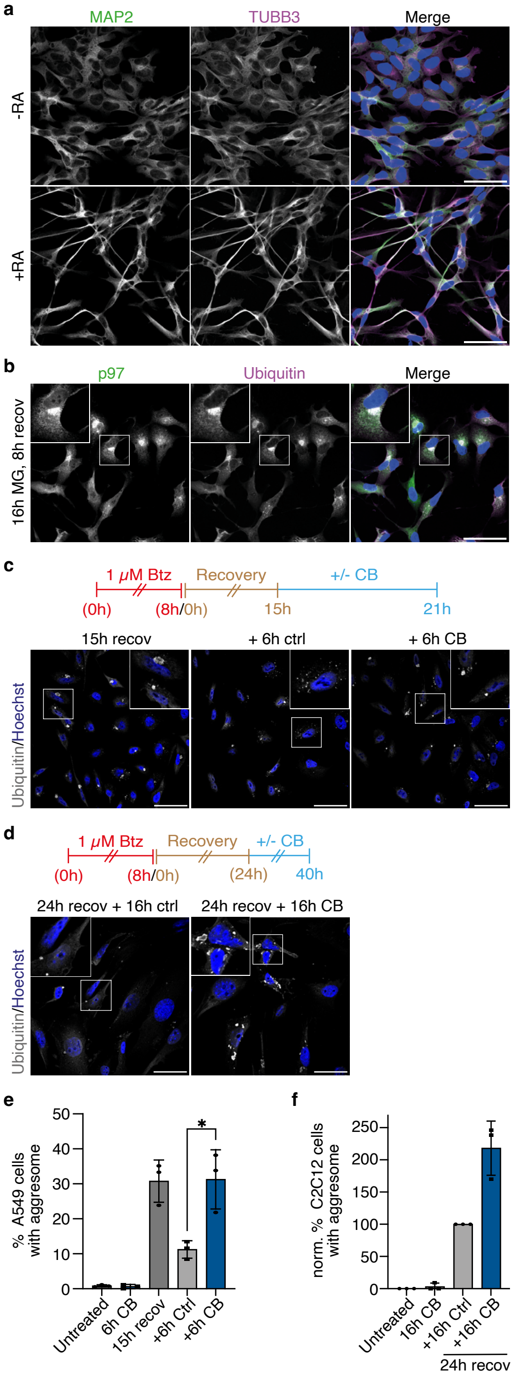

Supplementary Fig. 5 | Aggresome formation and clearance in additional cell lines.
a Differentiation state of SH-SY5Y cells. SH-SY5Y cells were cultured in the absence or presence of retinoic acid (RA) for 3 days prior to fixation, immunostaining against the neuronal marker proteins MAP2 and TUBB3 and analysis by confocal microscopy. Representative result from two independent experiments. **b** RA-differentiated SH-SY5Y cells treated with MG-132 (2.5 μM, 16h) were fixed after 8h recovery, followed by immunostaining against p97 and ubiquitin and analysis by confocal microscopy. Representative result from two independent experiments. **c** A549 cells were treated and analyzed exactly as the HeLa cells in Fig. 3a. Scale bars, 50 μm. **d** C2C12 cells were treated with Btz (1 μM, 8h) and recovered for 40h in the absence or presence of 0.5 μM CB-5083 in the last 16h prior to fixation, immunostaining against ubiquitin and analysis by confocal microscopy. Scale bars, 50 μm. **e** Quantification of the percentage of A549 cells with an aggresome in **c**. Shown is the mean ± SD from n = 3 biological replicates with ≥ 80 cells per condition and replicate. Unpaired, two-tailed Student's t-test. **f** Quantification of the percentage of C2C12 cells with an aggresome in **d**. Data were normalized to the percentage of C2C12 cells with an aggresome after 40h of recovery, which was set to 100 %. Shown is the mean ± SD from n = 3 biological replicates with ≥ 60 cells per condition and replicate.

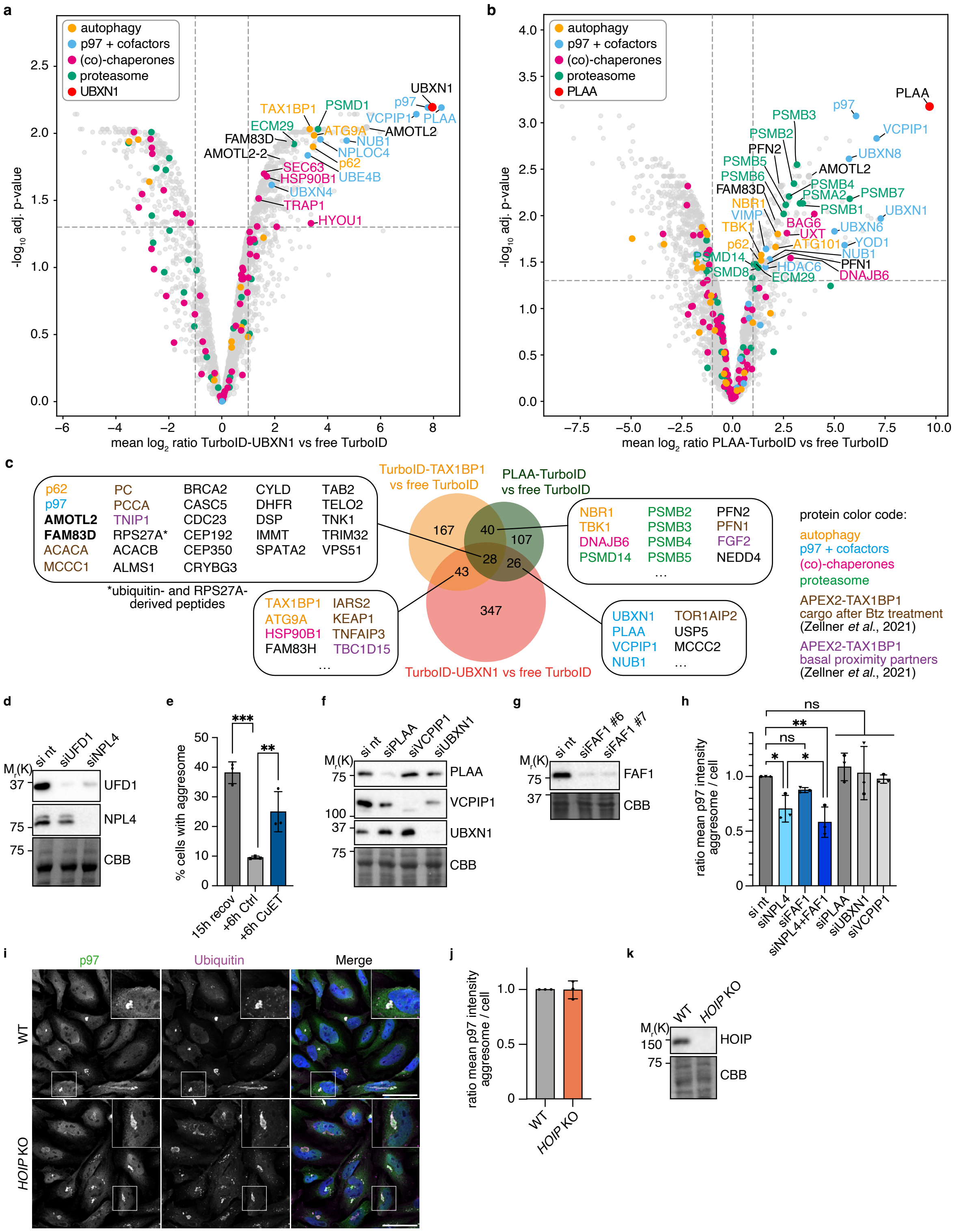

Supplementary Fig. 6 | Proximitomes of UBXN1 and PLAA and role of p97 cofactors in aggresome clearance.
a, b Volcano plots for HeLa cells expressing TurboID-UBXN1 (n = 3 biological replicates) (**a**) or PLAA-TurboID (n = 4 biological replicates) (**b**) versus free TurboID after Btz treatment followed by 21h recovery in the presence of 100 nM BafA1 for the last three hours and of 50 µM biotin for the last 20 min. Statistical testing, thresholds and color code as in Fig. 2cd. **c** Venn diagram highlighting the overlap between significantly enriched TurboID-TAX1BP1, TurboID-UBXN1 and PLAA-TurboID proximity partners (under aggrephagy condition, in comparison to free TurboID). Selected proteins are highlighted in the color code indicated to the right. **d** HeLa cells treated for 72h with siRNA targeting UFD1 or NPL4 were immunoblotted for the respective target protein to validate knockdown efficiency. **e** HeLa cells treated with Btz (1 µM, 8h) and fixed after 15h recovery, or recovered for six additional hours in the absence or presence of CuET, were immunostained against ubiquitin. The percentage of cells with aggresome was quantified; shown is the mean ± SD from n = 3 biological replicates with ≥ 100 cells per condition and replicate. One-way ANOVA. **f, g** As in **d**, but validating the knockdown of the indicated p97 cofactors. **h** p97 recruitment to aggresomes was analyzed in control cells (nt) and cells depleted of NPL4, FAF1 or NPL4 + FAF1 after Btz treatment and 15h recovery by confocal immunofluorescence microscopy using antibodies against p97 and ubiquitin. The mean p97 intensity at aggresomes was quantified and normalized to the mean p97 intensity of the respective whole cell. Shown is the mean ± SD from n = 3 biological replicates with ≥ 70 cells per condition and replicate. One-way ANOVA. To compare the siNPL4 sample to the siNPL4 + FAF1 sample, an unpaired, two-tailed Student's t-tests was performed. **i** p97 recruitment to aggresomes was analyzed in WT and *HOIP* KO cells treated as in **h**. Scale bars, 50 µm. (**j**) Quantification of **i** as described in **h**. Shown is the mean ± SD from n = 3 biological replicates with ≥ 100 cells per condition and replicate. **k** Lysates from WT and *HOIP* KO cells were immunoblotted for HOIP to validate KO efficiency. All immunoblots shown in this figure are representative for two independent experiments.

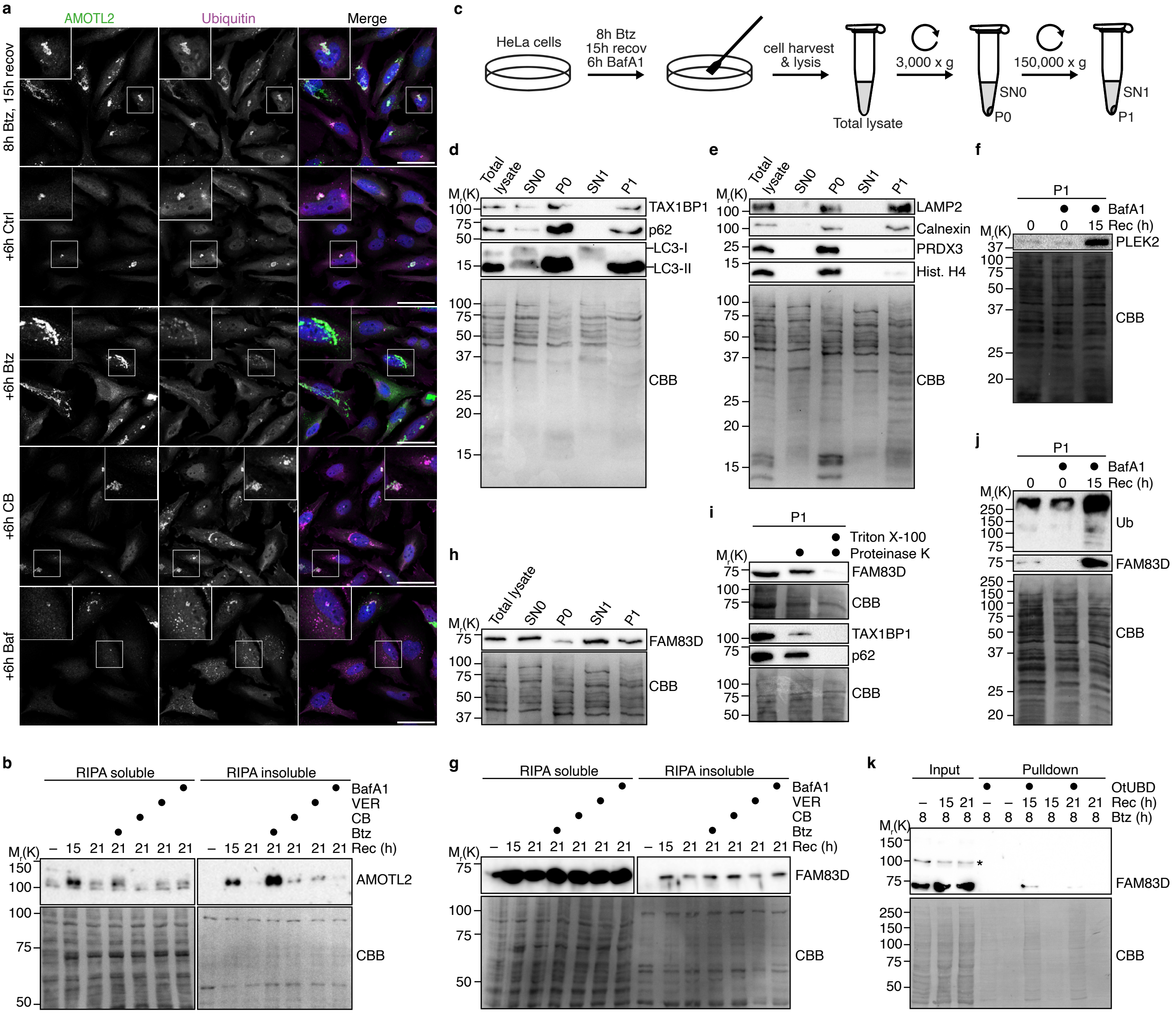

Supplementary Fig. 7 | Proteasomal degradation of AMOTL2 and presence of FAM83D in autophagosome-enriched fraction.
a HeLa cells treated with Btz (1 µM, 8h) and fixed after 15h recovery, or recovered for six additional hours in the absence or presence of 1 µM CB-5083, 100 nM BafA1 or 1 µM Btz, were immunostained against ubiquitin and AMOTL2. Scale bars, 50 µm. Representative result from three independent experiments. **b** HeLa cells treated with Btz (1 µM, 8h) and harvested after 15h recovery, or recovered for six additional hours in the absence or presence of 1 µM Btz, 1 µM CB-5083, 10 µM VER or 100 nM BafA1 were lysed in RIPA buffer. Untreated HeLa cells served as negative control. RIPA soluble and RIPA insoluble fractions were immunoblotted for AMOTL2; the Coomassie stained membrane served as loading control (CBB). **c** Scheme illustrating workflow to isolate autophagosome-enriched fraction P1. **d, e** Individual fractions from the autophagosome-enrichment protocol were immunoblotted for the autophagosome markers TAX1BP1, p62 and LC3 (**d**) or for marker proteins of other cell compartments (**e**), including LAMP2 (lysosomes), Calnexin (ER), PRDX3 (mitochondria) and Histone H4 (nuclei). **f** Autophagosome-enriched P1 fractions of HeLa cells that were left untreated, or treated for 6h with 100 nM BafA1 only, or treated for 6h with 100 nM BafA1 after 15h of recovery from 8h Btz treatment, were immunoblotted for PLEK2; the Coomassie stained membrane served as loading control (CBB). **g** Same as **b**, but RIPA soluble and RIPA insoluble fractions were immunoblotted for FAM83D. **h** Same as **d**, **e**, but fractions were immunoblotted for FAM83D. **i** Same as Fig. **6e**, but the P1 fractions were immunoblotted for FAM83D. **j** Same as **f**, but the P1 fractions were immunoblotted for ubiquitin (Ub) and FAM83D. **k** Same as Fig. **6a**, but input and pulldown samples were immunoblotted for FAM83D. An unspecific band in the input is marked with an asterisk. All immunoblots shown in this figure are representative for two independent experiments.

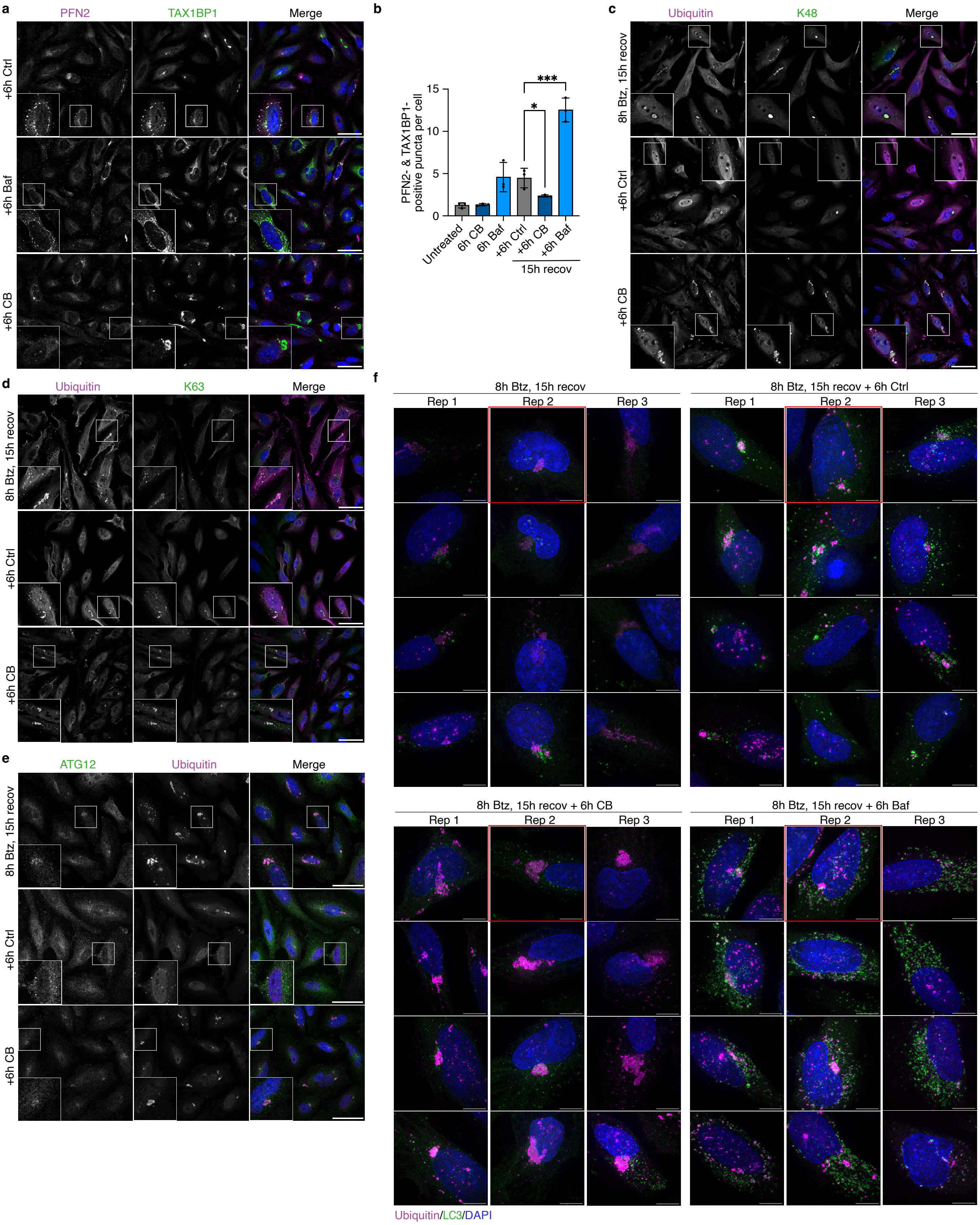

Supplementary Fig. 8 | Association of PFN2, ubiquitin, ATG12 and LC3 with aggresomes upon p97 inhibition.
a HeLa cells treated with Btz (1 µM, 8h) and recovered for 21h in the absence or presence of 100 nM BafA1 or 1 µM CB-5083 in the last 6h were immunostained against PFN2 and TAX1BP1. Scale bars, 50 µm. **b** Quantification of number of PFN2 and TAX1BP1 double-positive structures per cell in **a**. Shown is the mean ± SD from n = 3 biological replicates with ≥ 90 cells per condition and replicate. One-way ANOVA. **c, d** HeLa cells treated with Btz (1 µM, 8h) and fixed after 15h recovery, or recovered for six additional hours in the absence or presence of 1 µM CB-5083, were immunostained using an anti-ubiquitin antibody that recognizes all polyubiquitylated protein conjugates as well as an anti-ubiquitin antibody specifically recognizing K48-linked (**c**) or K63-linked (**d**) ubiquitin chains. Scale bars, 50 µm. Representative result from two independent experiments. **e** Same as **c**, but using antibodies against ubiquitin and ATG12. Scale bars. 50 µm. Representative result from two independent experiments. **f** HeLa cells treated with Btz (1 µM, 8h) were fixed after 15h recovery, or recovered for six additional hours in the absence or presence of 1 µM CB-5083 or 100 nM BafA1, and analyzed by 3D structured illumination microscopy using antibodies against ubiquitin and LC3. Shown are the maximum intensity projections from four representative cells per replicate (Rep) and condition. Cells chosen for 3D rendering shown in Fig. 6j are framed with a red box. Scale bars, 10 µm.

Supplementary Table 1

Materials used in this study

Reagent or resource	Source	Identifier
**Antibodies**		
Goat polyclonal anti-biotin-FITC (1:80 for IF)	Sigma-Aldrich	Cat# F6762
Mouse monoclonal anti-alpha-tubulin (1:2000 for WB)	Sigma-Aldrich	Cat# T5168, RRID: AB_477579
Rabbit polyclonal anti-VCP (1:300 for IF)	Bethyl Laboratories	Cat# A300-589A, RRID: AB_495512
Rabbit polyclonal anti-FAF1 (1:10,000 for WB)	Max Planck Institute of Biochemistry, animal facility	AB65
Mouse monoclonal anti-FAF1 (1:50 for IF)	Santa Cruz	Cat# sc-393965
Rabbit polyclonal anti-NPL4 (1:150 for IF; 1:1,000 for WB)	Sigma-Aldrich	Cat# HPA021560
Rabbit polyclonal anti-UFD1 (1:150 for IF; 1:1,000 for WB)	Proteintech	Cat# 10615
Rabbit polyclonal anti-PLAA (1:200 for IF; 1:1,000 for WB)	Sigma-Aldrich	Cat# HPA020996
Rabbit polyclonal anti-UBXN1 (1:200 for IF; 1:1,000 for WB)	Sigma-Aldrich	Cat# HPA012669
Mouse monoclonal anti-VCPIP1 (1:1,000 for WB)	Santa Cruz	Cat# sc-515291
Rabbit polyclonal anti-HOIP (1:150 for IF; 1:1,000 for WB)	Bethyl Laboratories	Cat# A303-560A-T
Rabbit polyclonal anti-DNAJB6 (1:200 for IF; 1:1,000 for WB)	Proteintech	Cat# 11707-1-AP
Mouse monoclonal anti-ubiquitin (1:350 for IF)	Enzo	Cat# ENZ-ABS840-0500
Mouse monoclonal anti-ubiquitin (1:2,000 for WB)	Enzo	Cat# BML-PW0930-0100
Rabbit monoclonal anti-ubiquitin Lys48 specific (1:400 for IF)	Sigma-Aldrich	Cat# 05-1307
Rabbit monoclonal anti-ubiquitin Lys63 specific (1:100 for IF)	Sigma-Aldrich	Cat# 05-1308
Rabbit polyclonal anti-LC3B (1:400 for IF)	MBL	Cat# PM036
Rabbit polyclonal anti-LC3B (1:1000 for WB)	Sigma-Aldrich	Cat# L7543
Rabbit monoclonal anti-LC3B (1:400 for IF)	Cell Signaling	Cat# 3868
Mouse monoclonal anti-WIPI2 (1:300 for IF)	Biorad	Cat# MCA5780GA
Rabbit monoclonal anti-vimentin (1:300 for IF)	Cell Signaling	Cat# 5741
Rabbit polyclonal anti-TAX1BP1 (1:150 for IF; 1:1,000 for WB)	Sigma-Aldrich	Cat# HPA024432

Rabbit polyclonal anti-p62 (1:2,500 for WB)	Sigma-Aldrich	Cat# P0067
Mouse monoclonal anti-p62 (1:300 for IF)	Santa Cruz	Cat# sc-28359
Rabbit polyclonal anti-NDP52 (1:100 for IF)	Proteintech	Cat# 12229
Rabbit polyclonal anti-NBR1 (1:100 for IF; 1:1,000 for WB)	Proteintech	Cat# 16004
Rabbit polyclonal anti-OPTN (1:100 for IF)	Proteintech	Cat# 10837
Mouse monoclonal anti-V5 (1:200 for IF)	Cell Signaling	Cat# E9H80
Mouse monoclonal anti-GAPDH (1:2,000 for WB)	UBPBio	Cat# Y1041
Rabbit polyclonal anti-AMOTL2 (1:200 for IF; 1:1,000 for WB)	Novus	Cat# NBP2-92875
Mouse monoclonal anti-PFN2 (1:100 for IF)	Proteintech	Cat# 60094-2-Ig
Rabbit polyclonal anti-FAM83D (1:100 for IF; 1:1,000 for WB)	Thermo Fisher Scientific	Cat# PA5-99011
Rabbit polyclonal anti-PLEK2 (1:100 for IF; 1:750 for WB)	Proteintech	Cat# 11685-1-AP
Mouse monoclonal anti-TUBB3 (1:100 for IF)	Proteintech	Cat# 66375-1-Ig
Rabbit polyclonal anti-MAP2 (1:100 for IF)	Proteintech	Cat# 17490-1-AP
Rabbit polyclonal anti-ATG12 (1:100 for IF)	Cell Signaling	Cat# 2010T
Rabbit monoclonal anti-p-p62 S403 (1:100 for IF; 1:1,000 for WB)	Cell Signaling	Cat# 39786
Rabbit monoclonal anti-p-p62 S349 (1:100 for IF; 1:1,000 for WB)	Cell Signaling	Cat# 16177
Rabbit monoclonal anti-p-TBK1 (1:100 for IF)	Cell Signaling	Cat# 5483
Rabbit polyclonal anti-TNIP1 (1:200 for IF)	Proteintech	Cat# 15104-1-AP
Rabbit polyclonal anti-PSMB5 (1:200 for IF)	Thermo Fisher Scientific	Cat# PA1-977
Rabbit polyclonal anti-PSMD14 (1:100 for IF)	Thermo Fisher Scientific	Cat# 38-0200
Mouse monoclonal anti-Hsp70 (1:100 for IF)	Santa Cruz	Cat# sc-32299
Rabbit polyclonal anti-Calnexin (1:1,000 for WB)	Abcam	Cat# ab22595
Rabbit polyclonal anti-Histone H4 (1:1,000 for WB)	Proteintech	Cat# 16047-1-AP
Mouse monoclonal anti-LAMP2 (1:1,000 for WB)	Novus	Cat# NBP2-22217
Mouse monoclonal anti-PRDX3 (1:10,000 for WB)	Proteintech	Cat# 66810-1

Goat polyclonal anti-Mouse IgG HRP (1:7,500 for WB)	Dianova (Jackson ImmunoResearch)	Cat# 115-035-003, RRID:AB_10015289
Goat polyclonal anti-Rabbit IgG HRP (1:7,500 for WB)	Dianova (Jackson ImmunoResearch)	Cat# 111-035-045, RRID: AB_2337938
Alexa Fluor 488 Goat Anti-Rabbit IgG (H+L) (1:500 for IF)	Thermo Fisher Scientific	Cat# A-11070, RRID: AB_142134
Alexa Fluor 488 Goat Anti-Mouse IgG (H+L) (1:500 for IF)	Thermo Fisher Scientific	Cat# A-11017, RRID: AB_143160
Alexa Fluor 568 Goat anti-Rabbit IgG (H+L) Cross-Adsorbed (1:500 for IF)	Thermo Fisher Scientific	Cat# A-11011, RRID:AB_143157
Alexa Fluor 594 Goat Anti-Rabbit IgG (H+L) (1:500 for IF)	Thermo Fisher Scientific	Cat# A-11072, RRID: AB_142057
Alexa Fluor 594 Goat Anti-Mouse IgG (H+L) (1:500 for IF)	Thermo Fisher Scientific	Cat# A-11020, RRID: AB_141974
Alexa Fluor 647 Goat Anti-Rabbit IgG (H+L) (1:400 for IF)	Thermo Fisher Scientific	Cat# A21246, RRID:AB_2535814
Alexa Fluor 647 Goat Anti-Mouse IgG (H+L) (1:400 for IF)	Thermo Fisher Scientific	Cat# A21236, RRID:AB_2535805
Goat polyclonal anti-biotin-FITC (1:80 for IF)	Sigma-Aldrich	Cat# F6762
Mouse monoclonal anti-alpha-tubulin (1:2000 for WB)	Sigma-Aldrich	Cat# T5168, RRID: AB_477579
**Bacterial strains**		
XL1 Blue	Stratagene	Cat# 200249
XL10 Gold	Agilent	Cat# 200516-4
DH5-Alpha	Thermo Fisher Scientific	Cat# 18265017
**Critical commercial resources**		
CB-5083	Selleckchem	Cat# S8101
MG-132	Selleckchem	Cat# S2619
Epoxomicin	Biomol	Cat# P7099d
Capzimin dimer	Bio-Techne	Cat# 6781/10
Tubacin	MedChemExpress	Cat# HY-13428
Tubastatin A	MedChemExpress	Cat# HY-13271
NMS-873	Sellekchem	Cat# S7285
Bafilomycin A1	Cayman Chemical	Cat# Cay11038-500
Bafilomycin A1	Sigma Aldrich	Cat# B1793-10mg
CuET	Sigma Aldrich	Cat# SML2675-100mg
Wortmannin	Cayman Chemical	Cat# Cay10010591-1
Bortezomib	Selleckchem	Cat# S1013
VER155008	Selleckchem	Cat# S7751
HOIPIN8	MedChemExpress	Cat# MCE-HY-122882
Cell-based Proteasome Glo-Assay	Promega	Cat# G8660
Strep-HRP Conjugate	Thermo Fisher Scientific	Cat# S911
Clarity Western ECL Substrate	Bio-Rad	Cat# 1705061
Immobilon-P Transfer Membrane (PVDF)	Merck Millipore	Cat# IPVH00010

cOmplete, EDTA-free Protease Inhibitor Cocktail	Roche	Cat# 04693132001
Phenylmethanesulfonyl fluoride (PMSF)	Carl Roth	Cat# 6367.1
Lipofectamine 2000	Thermo Fisher Scientific	Cat# 11668030
Ni-NTA Agarose	Qiagen	Cat# 30230
Glutathione-Sepharose-Beads	GE Healthcare	Cat# 17-0756-01
Opti-MEM	Thermo Fisher Scientific	Cat# 31985602
Neurobasal medium	Thermo Fisher Scientific	Cat# 21103-049
B27 supplement	Thermo Fisher Scientific	Cat#17504044
GlutaMAX	Thermo Fisher Scientific	Cat# 330550061
All-*trans*-retinoic acid (ATRA)	Thermo Fisher Scientific	Cat# 207341000
Doxycycline	Invivogen	Cat# ant-doxbl-05
Neomycin/G418	Invivogen	Cat# ant-gn-5
Poly-*D*-lysin	Thermo Fisher Scientific	Cat# A3890401
Pierce Strepatavidin magnetic beads	Thermo Fisher Scientific	Cat# 88817
Polybrene	Santa Cruz	Cat# sc-134220
Polyethylenimine (PEI)	Polysciences	Cat# 23966-1
DAPI (4',6-Diamidino-2-Phenylindole, Dihydrochloride)	Thermo Fisher Scientific	Cat# D1306
Marienfeld Precision cover glasses	VWR	Cat# 107032
16% Paraformaldehyde, Electron-Microscopy Grade	Electron Microscopy Science	Cat# 15710
Vectashield	Vector Laboratories	Cat# H-1000-10
ProLong Glass Antifade Mountant	Thermo Fisher Scientific	Cat# P36980
Puromycin	InvivoGen	Cat# ant-pr-1
Hygromycin	InvivoGen	Cat# ant-hg-5
Tetracyclin	Sigma Aldrich	Cat# 87128
ReadyBlue Protein Gel Stain	Sigma-Aldrich	Cat# RSB-1L
RIPA Lysis Buffer	Thermo Fisher Scientific	Cat# 89901
Oligofectamine™	Thermo Fisher Scientific	Cat# 12252011
Pierce™ BCA Protein Assay Kit	Thermo Fisher Scientific	Cat# 23225
Triton X-100	Carl Roth	Cat# 3051.2
**Experimental models: cell lines**		
HeLa	ATCC	Cat# CCL-2
HeLa Flp-in-TRex	Thomas U. Mayer lab	N/A
A549	ATCC	Cat# CCL-185
ARPE-19	ATCC	Cat# CRL-2302
C2C12	ATCC	Cat# CRL-1772
SH-SY5Y	ATCC	Cat# CRL-2266
HEK293T	ATCC	Cat# CRL-3216
HeLa TAX1BP1 KO cell pool	This study	N/A

HeLa TAX1BP1 KO clone A2	This study	N/A
HeLa TAX1BP1 KO clone A2 pINDUCER R-TAX1BP1-mScarlet	This study	N/A
HeLa DNAJB6 KO cell pool	This study	N/A
HeLa non-human-target cell pool	This study	N/A
$HeLa^{TMEM192\text{-}HA}$	ref. 1	N/A
$HeLa^{TMEM192\text{-}HA}$ ATG7 KO	ref. 1	N/A
HeLa TurboID-TAX1BP1	This study	N/A
HeLa Free TurboID	This study	N/A
HeLa Flp-in-TRex 6M&RRMmTDP43	This study	N/A
**Oligonucleotides**		
ON-TARGETplus human FAM104A siRNA - SMARTpool	Dharmacon	Cat# L-015015-02-0005
ON-TARGETplus Non-targeting pool	Dharmacon	Cat# D-001810-10-05
ON-TARGETplus human NPLOC4 siRNA - SMARTpool	Dharmacon	Cat# L-020796-01-0005
ON-TARGETplus human PLAA siRNA - SMART pool	Dharmacon	Cat# L-016215-00-0005
ON-TARGETplus human VCPIP1 siRNA - SMART pool	Dharmacon	Cat# L-019137-00-0005
ON-TARGETplus siRNA human UBXN1	Dharmacon	Cat# J-008652-12-0005
ON-TARGETplus siRNA human UFD1	Dharmacon	Cat# J-017918-06-0005
ON-TARGETplus siRNA human FAF1	Dharmacon	Cat# J-009106-06-0005
ON-TARGETplus siRNA human FAF1	Dharmacon	Cat# J-009106-07-0005
**Recombinant DNA**		
psPAX2	Addgene	Cat# 12260
pMD2.G	Addgene	Cat# 12259
pLentiCRISPRv2	Addgene	Cat# 52961
V5-TurboID-NES/pcDNA3	Addgene	Cat# 107169
pcDNA5 6M RRMm TDP43	ref. 2	N/A
pUC19	NEB	Cat# N3041S
pSpCas9(BB)-2A-Puro	Addgene	Cat# 48139
pcDNA5-FRT/TO	Life Technologies	pAB1952
pOG44	Life Technologies	pAB1953
pRRLSin.cPPT.PGK	AG Eilers (modified from Addgene Cat# 12252)	N/A
TAX1BP1 homology repair template pUC19	This study	pAB 3097
TAX1BP1 sgRNA1 in pSpCas9(BB)-2A-Puro	This study	pAB3086

FreeTurboID pRRLSin.cPPT.PGK	This study	pAB3100
Non human control sgRNA1 in pLentiCRISPRv2	Manuel Kaulich, University of Frankfurt	pAB2585
Non human control sgRNA2 in pLentiCRISPRv2	Manuel Kaulich, University of Frankfurt	pAB2586
Non human control sgRNA3 in pLentiCRISPRv2	Manuel Kaulich, University of Frankfurt	pAB2587
Non human control sgRNA4 in pLentiCRISPRv2	Manuel Kaulich, University of Frankfurt	pAB2588
TAX1BP1 sgRNA1 in pLentiCRISPRv2	This study	pAB3201
DNAJB6 sgRNA1 in pLentiCRISPRv2	This study	pAB3187
DNAJB6 sgRNA2 in pLentiCRISPRv2	This study	pAB3188
DNAJB6 sgRNA3 in pLentiCRISPRv2	This study	pAB3189
pInducer TAX1BP1-mScarlet	ref. 3	SMC2214
**Software**		
Fiji	http://fiji.sc	RRID: SCR_002285
Image Lab Software	Bio-Rad	RRID: SCR_014210
CellProfiler	https://cellprofiler.org/	RRID:SCR_007358
UCSF ChimeraX	https://www.cgl.ucsf.edu/chimerax/	RRID:SCR_015872
GraphPad Prism	https://www.graphpad.com/scientific-software/prism/	RRID:SCR_002798
g:Profiler	http://biit.cs.ut.ee/gprofiler/	RRID:SCR_006809
ApE	http://biologylabs.utah.edu/jorgensen/wayned/ape/	RRID:SCR_014266
**Primers, gRNAs and gblocks**	**Sequence**	
TAX1BP1_gRNA	CTGCAATGGGACTTCTTGAA	
DNAJB6_gRNA1	CTTTCCAAGATATCGGAAAC	
DNAJB6_gRNA2	TCTTTGGTAAGTTAATCACG	
DNAJB6_gRNA3	TTACGCCTTTTTAATATCCT	
Non-human-target-309-KO-1-R_156	AACATGACGTTCAAGATTGG	
Non-human-target-365-KO-5-R_5	ACCACTGTTCTACGCGCAGG	
Non-human-target-415-KO-2-R_24	TTGAACGGGCCGCGGAAGCG	
Non-human-target-42-KO-15-R_115	GTTTTCATGGAGTGATAATG	
TAX1BP1_PCRseq_fw	CCCAAGGGTTCTACATAGGAAG	
TAX1BP1_PCR-seq_rv	GTTAAGGTGTAATGACATTCCAGG	

TAX1BP1_gblock1	CGCCAGGGTTTTCCCAGTCACGACGTTGTAAAACGACGG<br>CCAGTGTTTTAAAGGCCCCTAATGATTCTAATGTGTAACCA<br>GGGTTGAGAAATGCTGGTCCAGGGGATTAGAAGGGCAGG<br>AATGTAGGCACAGAGATGTTAGCGTCAGGTTTAGTTATAG<br>GACCCCAAAACCTAGGGAGAGGGGAAGAGATGAATGGGA<br>TGGGCTAGGTTAGGGAGGAGAATGATGAAGCCCAAGGGT<br>TCTACATAGGAAGGAAATGTTAATTGACCCATAGCCAGTA<br>AAATACTGACTTTTAACTGTTCTTGTTTCTGTTTCTTAGTGT<br>TTGTTACTAGGAGCCAGCAGGTTTATATTGGCAGCGTGTG<br>CACAAAAACACCTCTGGTTCATTTGCTTTTGATTTAAATAG<br>AAGCAGAGGGCTAGGCTTTCCAGGAGAGTTAATTTTTGGT<br>TTTGGTTTTTTAACTTTTAAATGCCCATATAACTTTCTGAGC<br>CAAATGGGTTTTAGTTATTTTTCCAGTTTGTCTGTATTTTTAT<br>ATTTAATCAATTTTTAAGAAGTTCCAGTATTTACAAAGTATA<br>TATTTTCTTAATTATGACATGCAAATATTCATGAAACTTATAT<br>TAAATATTATGTATTTTCTGAAGGCATAAGTTTATTCTTAGT<br>TGGTATAATTTAACTTGTATGAATTACTTGTTTCAGATTCAC<br>AGCCACCATGGGCAAGCCCATCCCCAACCCCCTGCTGG<br>GCCTGGAC
TAX1BP1_gblock2	CGAAAAGCTGCAGCTGCCTCCCCTGGAGCGCCTGACCCT<br>GGGTAGCGGATCCGGGAGCACAAGTTTCCAAGAAGTTCC<br>TTTACAGACTTCCAACTTTGCCCATGTCATCTTTCAAAATG<br>TGGCCAAGAGTTACCTTCCTAATGCACACCTGGAATGTCA<br>TTACACCTTAACTCCATATATTCATCCACATCCAAAAGATT<br>GGGTTGGTATATTCAAGGTAAGAAAGCTTTCTGAATATGTT<br>CTCCATGTAAACACTTAAAATTTTCTTTTAAAACAGATTGAT<br>AAGTAGCTTTTACTTGCCTTAACTCTGCATTAAGACTCATTT<br>TTGTAAACATCACCATGAAATATTTAACAGTGGGGAGTAGA<br>TAAAATGGATGTTGGGAACTCCATTTCACCAGATTTTCCTC<br>TCCCTGATCAGAAAGTTTACCTTCACTTAAGGACTTGTGAA<br>AATTTTTTCAGAATCTTCTTTTCCTATTTTCTCCATTCATTAC<br>CTGCAATAAAAAAAGATCTTTTTCTTTGGGTTCTTATATTAT<br>TTACCATTTTTTCCAGTCATTTGAAATTAATTTTATACAGCTT<br>GACAGCCATGTTATTATCCCTTCTACTGTCCTGCATGGCTT<br>AAATATTGCTCAGATTGTATTACACAGTTTTAAGAGTATTCT<br>GTTTGTATTACACAGTTTTAAAGTATTCTGTTTAACATGACT<br>GAATAGTTGGCGTAATCATGGTCATAGCTGTTTCCTGTGT<br>GAAATTG

Supplementary References

1 Eapen, V. V., Swarup, S., Hoyer, M. J., Paulo, J. A. & Harper, J. W. Quantitative proteomics reveals the selectivity of ubiquitin-binding autophagy receptors in the turnover of damaged lysosomes by lysophagy. *Elife* **10**, doi:10.7554/eLife.72328 (2021).

2 Perez-Berlanga, M. *et al.* Loss of TDP-43 oligomerization or RNA binding elicits distinct aggregation patterns. *EMBO J* **42**, e111719, doi:10.15252/embj.2022111719 (2023).

3 Bauer, B., Idinger, J., Schuschnig, M., Ferrari, L. & Martens, S. Recruitment of autophagy initiator TAX1BP1 advances aggrephagy from cargo collection to sequestration. *EMBO J* **43**, 5910-5940, doi:10.1038/s44318-024-00280-5 (2024).
