# Supplementary material for: p97/VCP is required for piecemeal autophagy of aggresomes
Source: Nat Commun. 2025 May 7;16:4243. doi: 10.1038/s41467-025-59556-x (PMC12059050; doi:10.1038/s41467-025-59556-x)
Supplement: Supplementary file 2 — Description of Additional Supplementary Files [file 41467_2025_59556_MOESM2_ESM.pdf]

Description of Additional Supplementary Files:

Supplementary Data 1: Complete results of the TurboID proximity proteomics experiments shown in Fig. 2cd and Supplementary Fig. 6ab.

Supplementary Data 2: Complete results of the GO term analysis shown in Supplementary Fig. 3de.

Supplementary Movie 1: Animation of the representative 3D rendering shown in Figure 7j for the 8h Btz, 15h recovery condition.

Supplementary Movie 2: Animation of the representative 3D rendering shown in Figure 7j for the + 6h control condition.

Supplementary Movie 3: Animation of the representative 3D rendering shown in Figure 7j for the + 6h Baf condition.

Supplementary Movie 4: Animation of the representative 3D rendering shown in Figure 7j for the + 6h CB condition.
