# Supplementary material for: p97/VCP is required for piecemeal autophagy of aggresomes
Source: Nat Commun. 2025 May 7;16:4243. doi: 10.1038/s41467-025-59556-x (PMC12059050; doi:10.1038/s41467-025-59556-x)
Supplement: Supplementary file 9 — Reporting Summary [file 41467_2025_59556_MOESM9_ESM.pdf]

nature portfolio

Corresponding author(s): Prof. Dr. Alexander Buchberger

Last updated by author(s): Apr 15, 2025

Reporting Summary

Nature Portfolio wishes to improve the reproducibility of the work that we publish. This form provides structure for consistency and transparency in reporting. For further information on Nature Portfolio policies, see our Editorial Policies and the Editorial Policy Checklist.

Statistics

For all statistical analyses, confirm that the following items are present in the figure legend, table legend, main text, or Methods section.

n/a	Confirmed	
[ ]	[x]	The exact sample size (*n*) for each experimental group/condition, given as a discrete number and unit of measurement
[ ]	[x]	A statement on whether measurements were taken from distinct samples or whether the same sample was measured repeatedly
[ ]	[x]	The statistical test(s) used AND whether they are one- or two-sided<br>*Only common tests should be described solely by name; describe more complex techniques in the Methods section.*
[x]	[ ]	A description of all covariates tested
[ ]	[x]	A description of any assumptions or corrections, such as tests of normality and adjustment for multiple comparisons
[ ]	[x]	A full description of the statistical parameters including central tendency (e.g. means) or other basic estimates (e.g. regression coefficient) AND variation (e.g. standard deviation) or associated estimates of uncertainty (e.g. confidence intervals)
[ ]	[x]	For null hypothesis testing, the test statistic (e.g. *F*, *t*, *r*) with confidence intervals, effect sizes, degrees of freedom and *P* value noted<br>*Give P values as exact values whenever suitable.*
[x]	[ ]	For Bayesian analysis, information on the choice of priors and Markov chain Monte Carlo settings
[x]	[ ]	For hierarchical and complex designs, identification of the appropriate level for tests and full reporting of outcomes
[x]	[ ]	Estimates of effect sizes (e.g. Cohen's *d*, Pearson's *r*), indicating how they were calculated

Our web collection on statistics for biologists contains articles on many of the points above.

Software and code

Policy information about availability of computer code

Data collection	Microscopy: TCS SP2 equipped with an acousto optical beam splitter (Leica), TCS SP8 equipped with an acousto optical beam splitter (Leica), CSU-W1 spinning disk confocal (Yokogawa) on a Eclipse Ti-E motorized microscope (Nikon) equipped with a LUN-F XL solid state laser combiner (Nikon), Operetta CLS High-Content Imaging System (PerkinElmer)<br>Immunoblot: Gel Doc XR+system (Bio-Rad)<br>Luminescence measurement: Infinite 200 (Tecan)<br>Massspectrometry: nano-HPLC-ESI-MS/MS on an Orbitrap Elite (Thermo Fisher) or Q Exactive instrument (Thermo Fisher) in conjunction with UltiMate 3000 RSLCnano HPLC systems (Thermo Fisher)
Data analysis	Fiji (AggreCount macro, customized batch colocalization analysis macro), Microsoft Excel, GraphPad PRISM, ImageLab, CellProfiler, MaxQuant, Python, Harmony High-Content and Imaging Analysis Software (PerkinElmer)

For manuscripts utilizing custom algorithms or software that are central to the research but not yet described in published literature, software must be made available to editors and reviewers. We strongly encourage code deposition in a community repository (e.g. GitHub). See the Nature Portfolio guidelines for submitting code & software for further information.

Data

Policy information about availability of data

All manuscripts must include a data availability statement. This statement should provide the following information, where applicable:

- Accession codes, unique identifiers, or web links for publicly available datasets
- A description of any restrictions on data availability
- For clinical datasets or third party data, please ensure that the statement adheres to our policy

The data supporting the findings of this study are available within the article and its supplementary information files. Source Data underlying any reported means/averages in box plots, bar charts and tables as well as uncropped and unprocessed scans of all blots and gels including all quantified replicates are provided with this paper. Mass spectrometric raw data and MaxQuant result files have been deposited to the ProteomeXchange Consortium via the PRIDE partner repository and are accessible using the dataset identifier PXD053581.

Research involving human participants, their data, or biological material

Policy information about studies with human participants or human data. See also policy information about sex, gender (identity/presentation), and sexual orientation and race, ethnicity and racism.

Reporting on sex and gender	Key experiments of this study were performed in mammalian cell lines derived from female (HeLa, SH-SY5Y) and male (ARPE-19, A549) individuals and found to yield highly consistent results across the cell lines tested.
Reporting on race, ethnicity, or other socially relevant groupings	N/A
Population characteristics	N/A
Recruitment	N/A
Ethics oversight	N/A

Note that full information on the approval of the study protocol must also be provided in the manuscript.

Field-specific reporting

Please select the one below that is the best fit for your research. If you are not sure, read the appropriate sections before making your selection.

☒ Life sciences ☐ Behavioural & social sciences ☐ Ecological, evolutionary & environmental sciences

For a reference copy of the document with all sections, see nature.com/documents/nr-reporting-summary-flat.pdf

Life sciences study design

All studies must disclose on these points even when the disclosure is negative.

Sample size	The figure legends state how many biological replicates (N) were performed for each experiment. For the quantification of each experiment, at least three independent biological replicates were used. The number of cells (n) per biological replicate is also depicted in the figure legends.
Data exclusions	No data were excluded.
Replication	Quantification of biological replicates have been analyzed statistically. All replications were successfull.
Randomization	Randomization was not applied in this study.
Blinding	Threshold-based automated image analysis was performed to avoid biased analysis.

Reporting for specific materials, systems and methods

We require information from authors about some types of materials, experimental systems and methods used in many studies. Here, indicate whether each material, system or method listed is relevant to your study. If you are not sure if a list item applies to your research, read the appropriate section before selecting a response.

Materials & experimental systems

n/a	Involved in the study
☐	☒ Antibodies
☐	☒ Eukaryotic cell lines
☒	☐ Palaeontology and archaeology
☒	☐ Animals and other organisms
☒	☐ Clinical data
☒	☐ Dual use research of concern
☒	☐ Plants

Methods

n/a	Involved in the study
☒	☐ ChIP-seq
☒	☐ Flow cytometry
☒	☐ MRI-based neuroimaging

Antibodies

Antibodies used

Goat polyclonal anti-biotin-FITC Sigma-Aldrich Cat# F6762
Mouse monoclonal anti-alpha-tubulin Sigma-Aldrich Cat# T5168, RRID: AB_477579
Rabbit polyclonal anti-VCP Bethyl Laboratories Cat# A300-589A, RRID: AB_495512
Rabbit polyclonal anti-FAF1 Max Planck Institute of Biochemistry, animal house AB65
Mouse monoclonal anti-FAF1 Santa Cruz Cat# sc-393965
Rabbit polyclonal anti-NPL4 Sigma-Aldrich Cat# HPA021560
Rabbit polyclonal anti-UFD1 Proteintech Cat# 10615
Rabbit polyclonal anti-PLAA Sigma-Aldrich Cat# HPA020996
Rabbit polyclonal anti-UBXN1 Sigma-Aldrich Cat# HPA012669
Mouse monoclonal anti-VCPIP1 Santa Cruz Cat# sc-515291
Rabbit polyclonal anti-HOIP Bethyl Laboratories Cat# A303-560A-T
Rabbit polyclonal anti-DNAJB6 Proteintech Cat# 11707-1-AP
Mouse monoclonal anti-ubiquitin Enzo Cat# ENZ-ABS840-0500
Mouse monoclonal anti-ubiquitin Enzo Cat# BML-PW0930-0100
Rabbit monoclonal anti-ubiquitin Lys48 specific Sigma-Aldrich Cat# 05-1307
Rabbit monoclonal anti-ubiquitin Lys63 specific Sigma-Aldrich Cat# 05-1308
Rabbit polyclonal anti-LC3B MBL Cat# PM036
Rabbit polyclonal anti-LC3B Sigma-Aldrich Cat# L7543
Rabbit monoclonal anti-LC3B Cell Signaling Cat# 3868
Mouse monoclonal anti-WIPI2 Biorad Cat# MCA5780GA
Rabbit monoclonal anti-vimentin Cell Signaling Cat# 5741
Rabbit polyclonal anti-TAX1BP1 Sigma-Aldrich Cat# HPA024432
Rabbit polyclonal anti-p62 Sigma-Aldrich Cat# P0067
Mouse monoclonal anti-p62 Santa Cruz Cat# sc-28359
Rabbit polyclonal anti-NDP52 Proteintech Cat# 12229
Rabbit polyclonal anti-NBR1 Proteintech Cat# 16004
Rabbit polyclonal anti-OPTN Proteintech Cat# 10837
Mouse monoclonal anti-V5 Cell Signaling Cat# E9H80
Rabbit polyclonal anti-AMOTL2 Novus Cat# NBP2-92875
Mouse monoclonal anti-PFN2 Proteintech Cat# 60094-2-Ig
Rabbit polyclonal anti-PFN2 Sigma-Aldrich Cat# P0101
Rabbit polyclonal anti-FAM83D Thermo Fisher Scientific Cat# PA5-99011
Rabbit polyclonal anti-PLEK2 Proteintech Cat# 11685-1-AP
Mouse monoclonal anti-TUBB3 Proteintech Cat# 66375-1-lg
Rabbit polyclonal anti-MAP2 Proteintech Cat# 17490-1-AP
Rabbit polyclonal anti-TELO2 Sigma-Aldrich Cat# HPA041473
Rabbit polyclonal anti-ATG12 Cell Signaling Cat# 2010T
Rabbit monoclonal anti-p-p62 S403 Cell Signaling Cat# 39786
Rabbit monoclonal anti-p-p62 S349 Cell Signaling Cat# 16177
Rabbit polyclonal anti-p-p62 S272 Phosphosolutions Cat# p196-269
Rabbit monoclonal anti-p-TBK1 Cell Signaling Cat# 5483
Rabbit polyclonal anti-TNIP1 Proteintech Cat# 15104-1-AP
Rabbit polyclonal anti-PSMB5 Thermo Fisher Scientific Cat# PA1-977
Rabbit polyclonal anti-PSMD14 Thermo Fisher Scientific Cat# 38-0200
Mouse monoclonal anti-Hsp70 Santa Cruz Cat# sc-32299
Rabbit polyclonal anti-Calnexin Abcam Cat# ab22595
Rabbit polyclonal anti-Histone H4 Proteintech Cat# 16047-1-AP
Mouse monoclonal anti-LAMP2 Novus Cat# NBP2-22217
Mouse monoclonal anti-PRDX3 Proteintech Cat# 66810-1
Goat polyclonal anti-Mouse IgG HRP Dianova (Jackson ImmunoResearch) Cat# 115-035-003, RRID:AB_10015289
Goat polyclonal anti-Rabbit IgG HRP Dianova (Jackson ImmunoResearch) Cat# 111-035-045, RRID: AB_2337938
Alexa Fluor 488 Goat Anti-Rabbit IgG (H+L) Thermo Fisher Scientific Cat# A-11070, RRID: AB_142134

	Alexa Fluor 488 Goat Anti-Mouse IgG (H+L) Thermo Fisher Scientific Cat# A-11017, RRID: AB_143160<br>Alexa Fluor 568 Goat anti-Rabbit IgG (H+L) Cross-Adsorbed Thermo Fisher Scientific Cat# A-11011, RRID:AB_143157<br>Alexa Fluor 594 Goat Anti-Rabbit IgG (H+L) Thermo Fisher Scientific Cat# A-11072, RRID: AB_142057<br>Alexa Fluor 594 Goat Anti-Mouse IgG (H+L) Thermo Fisher Scientific Cat# A-11020, RRID: AB_141974<br>Alexa Fluor 647 Goat Anti-Rabbit IgG (H+L) Thermo Fisher Scientific Cat# A21246, RRID:AB_2535814<br>Alexa Fluor 647 Goat Anti-Mouse IgG (H+L) Thermo Fisher Scientific Cat# A21236, RRID:AB_2535805
Validation	The custom anti-FAF1 antibody (Rabbit polyclonal anti-FAF1, AB65, Max Planck Institute of Biochemistry animal facility) was knockout validated by immunoblotting and immunofluorescence microscopy. All other antibodies are commercially available (see company and catalog numbers above) and have been validated by the manufacturer for the applications used in this study.

Eukaryotic cell lines

Policy information about cell lines and Sex and Gender in Research

Cell line source(s)	HeLa (f), ATCC Cat# CCL-2; A549 (m), ATCC Cat# CCL-185; ARPE-19 (f), ATCC Cat# CRL-2302; SH-SY5Y (f), ATCC Cat# CRL-2266; HEK293T (f), ATCC Cat# CRL-3216; C2C12, ATCC Cat# CRL-1772; HeLa Flp-in-TRex, Thomas U. Mayer lab, University of Constance
Authentication	None of the cell lines used were authenticated.
Mycoplasma contamination	All cell lines tested negative for mycoplasma contamination.
Commonly misidentified lines<br>(See ICLAC register)	N/A

Plants

Seed stocks	N/A
Novel plant genotypes	N/A
Authentication	N/A
